# Effect of intraarticular inoculation of mesenchymal stem cells in dogs with hip osteoarthritis by means of objective force platform gait analysis: concordance with numeric subjective scoring scales

**DOI:** 10.1186/s12917-016-0852-z

**Published:** 2016-10-07

**Authors:** Jose M. Vilar, Belen Cuervo, Monica Rubio, Joaquín Sopena, Juan M. Domínguez, Angelo Santana, Jose M. Carrillo

**Affiliations:** 1 Department of Animal Pathology, Universidad de Las Palmas de Gran Canaria, Trasmontaña S/N, 35416 Arucas Las Palmas, Spain; 2 Department of Animal Medicine and Surgery, Cátedra Garcia Cugat, CEU Cardenal Herrera University, C/Tirant lo Blanc, 7, 46115 Alfara del Patriarca Valencia, Spain; 3 Department of Animal Medicine and Surgery, University of Cordoba, 14071 Cordoba, Spain

**Keywords:** Mesenchymal stem cells, Force platform, Visual analog scale, Pain assessment, Osteoarthritis, Dog

## Abstract

**Background:**

Subjective pain assessment scales have been widely used for assessing lameness in response to pain, but the accuracy of these scales has been questioned. To assess scale accuracy, 10 lame, presa Canario dogs with osteoarthritis (OA) associated with bilateral hip dysplasia were first treated with mesenchymal stem cells. Then, potential lameness improvement was analyzed using two pain scales (Bioarth and visual analog scale). These data were compared with similar data collected using a force platform with the same animals during a period of 6 months after treatment.

**Results:**

The F test for intraclass correlation showed that concordance in pain/lameness scores between the 2 measuring methodologies was not significant (*P* value ≥ 0.9213; 95 % confidence interval, –0.56, 0.11). Although subjective pain assessment showed improvement after 6 months, force platform data demonstrated those same animals had returned to the initial lameness state.

**Conclusion:**

Use of pain assessment scales to measure lameness associated with OA did not have great accuracy and concordance when compared with quantitative force platform gait analysis.

**Electronic supplementary material:**

The online version of this article (doi:10.1186/s12917-016-0852-z) contains supplementary material, which is available to authorized users.

## Background

Intensity of pain is difficult to accurately assess in dogs. Veterinarians assess the severity of pain in their patients using scoring systems based on several signs, including patient vocalization, activity level, degree of lameness, and reaction to manipulation. However, all of these signs are subjective and may be influenced by a variety of external factors [1].

Historically, 3 categories of subjective pain scoring systems have been used in veterinary medicine to assess pain severity and enable investigators to compare different therapeutic strategies: behavioral response (e.g., vocalization), physiologic indices (e.g., heart rate), and visual reports. Some of the most commonly used subjective scoring systems to assess pain include visual analog scales (VAS), numeric rating scales (NRS) and simple descriptive scales, all of which are based on behavioral signs [2]. Investigators have also attempted various combinations of these categories in an effort to devise a more complete pain scoring method (e.g., University of Melbourne Pain Scale) [3].

The usefulness of physiologic variables (heart rate, respiratory rate, blood pressure, pupil dilation, and serum epinephrine, norepinephrine, b-endorphin, and cortisol levels) to measure pain is limited because they measure not only the patients’ level of pain, but also their level of stress, fear and sedation, and can be affected by certain health conditions [4–7]. In addition, recent studies have shown that physiologic variables have no correlation with VAS reports or combination pain scores. To date, no single, readily acquired physiologic measurement has been consistently established as the standard for pain severity detection in cats or dogs [8–10].

In dogs, lameness scores depend upon visual observation of gait. Using a VAS, limb function is determined by marking points on a line scale, with 1 end of the line representing clinical normality (soundness) and the other end representing maximum lameness (i.e., non-weight bearing). On the other hand, NRS can also quantify certain characteristics (signs) that can define pain and/or lameness. These characteristics are classified in 4−5 descriptive categories as crepitation on mobilization, degree of muscle atrophy, functional limitation, range of movement, etc., [11].

Specifically, VAS is a unidimensional, subjective scale based on the level of pain intensity shown by the patient. It has been widely used for the assessment of sensorial intensity, experimentally-induced pain, and the mechanisms and efficacy of pharmacologic and non-pharmacologic treatments [12, 13].

The Bioarth scale is based on an NRS system and consists of 2 parts: 1 that scores radiological evidence of osteoarthritis (OA), and a second part that evaluates joint functionality by scoring functional limitation, articular mobility, and muscular atrophy [14].

Regardless of the scale methodology used in the field of veterinary medicine, the main disadvantage of scale systems is that the person who performs the evaluation of pain is the owner and/or the veterinarian. This introduces a variable, i.e. the observer, which can significantly alter pain scores [15] [see Additional file 1].

Based on these premises, some degree of variability exists in subjective assessments performed by an individual or group. This fact makes the interpretation of NRS or VAS results a challenge for investigators and clinicians [16].

Kinetic gait analysis has become an accepted technique for accurate and objective evaluation of limb function in humans and animals [17, 18]; for that reason, force platform gait analysis can be used as a tool to determine pain, disease, and healing of different units of the locomotor system [19–21]. In the same manner, this device is consistently used as an accurate, objective method to document the efficacy of different medical treatments of OA in dogs [22–25].

With a force platform, limb function is commonly analyzed measuring the peak vertical force (PVF, maximal force applied during stance phase) and vertical impulse (VI, total force applied over time) to quantitatively assess the degree of lameness [26–28].

In the field of regenerative veterinary medicine, autologous mesenchymal stem cell (MSC) therapy is a rapidly growing area of research. Stem cells have been shown to have an affinity for damaged joint tissue. In addition, recent in vivo studies have confirmed that stem cells have the ability to participate in the repair of damaged joint structures, including cruciate ligaments, menisci, and cartilage lesions [29]. However, several studies in MSC treatment of dogs with hip OA have reported different outcomes regarding duration of effect [14, 30, 31]. In those investigations, study designs varied, and animals of different conformations were examined.

The objective of this study was to evaluate the concordance between subjective and objective measures of limb function in dogs of the same breed that had OA lameness due to bilateral hip dysplasia and were treated with autologous MSCs. It was hypothesized that results obtained using the Bioarth and VAS scales, on the 1 hand, and PVF and VI analysis, on the other hand, would correlate inconsistently.

## Methods

### Animals

Ten adult, client-owned presa Canario dogs (6 males, 4 females) with lameness and pain attributed to OA associated with hip dysplasia were included in the study. A control group consisted of 5 sound and healthy dogs of the same breed. The sample size was selected based on the availability of the subjects of the same breed, same pathology and similar degree of severity in order to achieve a study group with maximal homogeneity. None of the dogs were forced to perform physical activity.

**Fig. 1:**
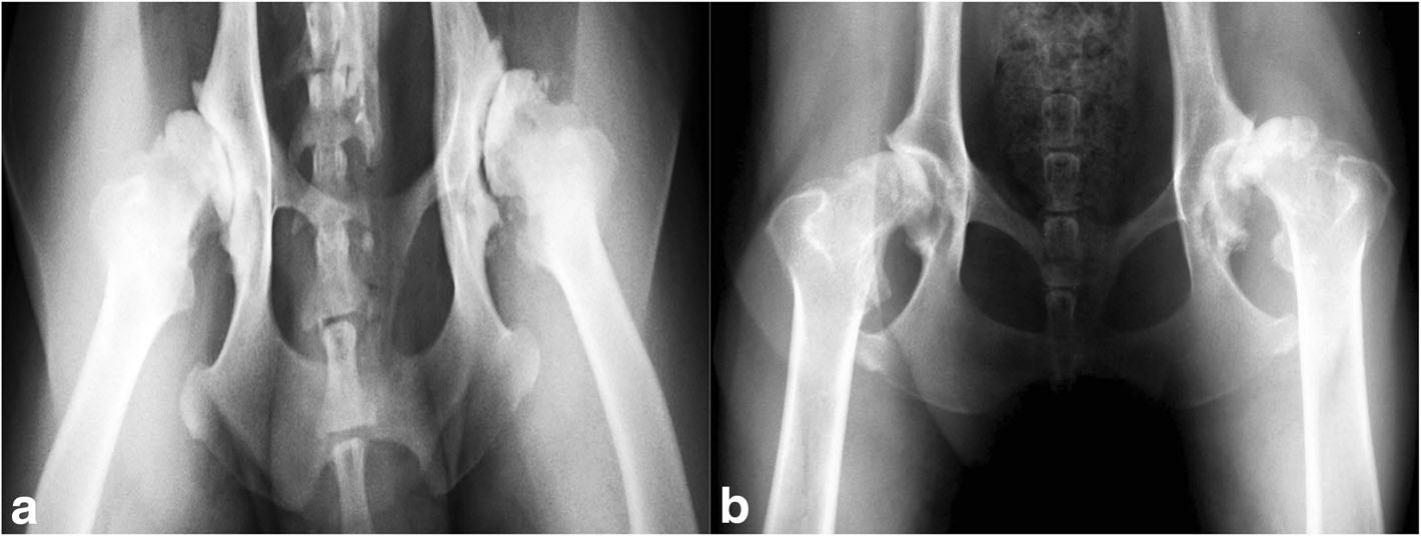
Radiographs showing lesions corresponding with D-degree (**a**) and E-degree (**b**) hip dysplasia

Ventrodorsal radiographs of the hips were performed under sedation with intravenous (i.v.) dexmedetomidine 0.05 mg (dexdomitor, Esteve, Barcelona, Spain) and analgesia with intramuscular (i.m.) butorphanol 0.05 mg/kg (torbugesic, Pfizer, Madrid, Spain). The obtained images confirmed the presence of OA compatible with D and E degrees of hip dysplasia as defined by the Fédération Cynologique Internationale (World Canine Organization). All D-degree dysplastic dogs showed obvious deviation from the norm with evidence of a shallow acetabulum, flattened femoral head, poor joint congruency, and in some cases, subluxation with marked changes of the femoral head and neck. All E-degree dysplastic dogs showed complete dislocation of the hip and severe flattening of the acetabulum and femoral head (Fig. 1a and b) [32].

Additional radiographs of knee and elbow joints and the lumbosacral region were taken after physical, orthopedic, and neurologic examinations were performed to ensure that hip OA was the main reason for the observed clinical signs in the study group and that general health was otherwise normal.

### Extraction and culture of MSCs

Stem cell extraction was performed under premedication with a combination of i.m. buprenorphine 0.01 mg/kg (Buprex, RB Pharmaceuticals, Bogotá, Colombia) and acepromazine 0.05 mg/kg i.m. (Equipromacina, Faltro ibérica, Barcelona, Spain). General anesthesia was induced with i.v. propofol 3 mg/kg (Vetofol, Esteve, Barcelona, Spain) and maintained with sevoflurane (SevoFlo, Abbott, Madrid, Spain). Patients were positioned in decubitus supinus. A biopsy of 20 g subcutaneous fat tissue (4–5 cm^3^) was collected from the inguinal region through a small surgical incision, and 120 mL blood was isolated under aseptic conditions and processed with the Dog Stem kit according to the manufacturer’s instructions. The incision was sutured with a simple, interrupted pattern. Meloxicam 0.1 mg/kg q 24 h PO (Metacam, Boehringer Ingelheim, Barcelona, Spain) was administered for 3 days post-surgery. Immediately after sample collection, fat biopsy and blood (in an anti-coagulant container) were maintained at 4 °C and sent for cell isolation and amplification under current GMP conditions to the Fat-Stem Laboratory (Belgium). The fat was processed with collagenase, and the cells were concentrated by centrifugation; the cells were then cultured in a bioreactor with controlled temperature and O^2^ and CO^2^ concentrations. Quality control was ensured by evaluating cell markers, sterility tests, and viability counts. Two weeks after biopsy, the Fat-Stem Laboratory returned the cultivated cells in two 2-mL, certified tubes containing 15 million adipose MSCs per tube.

### Inoculation of MSCs

The adult MSCs were infiltrated aseptically into the hip joints of the study dogs through conventional arthrocentesis sites. For this phase, the dogs were sedated with the same protocol used to take the radiographs. Prior to inoculation, fur was clipped in the articular region for both groups (study and control) in order to preserve the blindness of the study. The needle was introduced just cranioproximal to the trochanter major, aimed slightly ventrally and caudally. The appearance of joint fluid confirmed proper needle placement [33]. Once the excessive synovial liquid was drained, the MSCs were injected. Owners were advised to use meloxicam for pain management at home, if needed.

### Force platform gait analysis

Gait analysis was performed using a single platform mounted in the center of, and level with, a 7-m runway covered by a rubber mat. The mat weight was discarded by setting the tare button to “0 force” after the platform was covered. Dogs were leash guided at walk over the force platform by the same handler. Walk velocity was measured by use of a motion sensor (PS-2103a, Pasco, CA, USA) positioned 1 m from the platform. This device allowed the handler to ensure that animals walked homogeneously within a narrow variation of velocity (1.6 ± 0.5 m/s) and acceleration (≤ ± 0.5 m/s^2^).

Five valid trials, at a sampling frequency of 250 Hz, were obtained for each dog by an investigator blinded to the study (JMV). A trial was considered valid when the limb fully contacted the force platform at the approximate center, with the dog walking next to the handler without pulling on the leash. The trial was discarded if the dog appeared distracted during the measurement, if the limb struck the edge of the force plate, or if any portion of the contralateral paw hit the force plate. The platform was interfaced with a dedicated computer using DataStudio (Pasco, CA, USA), software specifically designed for the acquisition, numerical conversion, and storage of data. Both affected limbs were recorded at day 0, 30, 90, and 180 post-treatment. Finally, the obtained PVF and VI values were normalized relative to body weight (%).

Although each dog had bilateral lameness, only the measurements obtained from the more lame limbs (lesser PVF) were considered reliable, in order to avoid a possible bias caused by inconsistent weight redistribution to the less affected contra-lateral hindlimb. Only data from the more lame limbs were statistically compared using the Bioarth and VAS scales. The subjective methods used did not discriminate between limbs, and potential gait anomalies evaluated by the observer were recorded for the limb whereby lameness was more evident.

### Subjective scales

#### VAS

VAS was graphed using a horizontal line, 100 mm in length, anchored by word descriptors at each end: “NO PAIN” on the left and “WITHOUT SUPPORT” on the right. An experienced clinician blinded to treatment group (JMC) marked on the line the points at which himself and the client/owner felt represented the current lameness state of the dog. The VAS score was then determined by measuring in millimeters from the left end of the line to the mean value given by clinician and dog owner scores. The obtained results were expressed in percentages. A higher value means more severe lameness.

#### Bioarth assessment scale

Functional assessment using the Bioarth assessment scale evaluates the 3 basic functional parameters: functional limitation, joint mobility, and muscle atrophy. For functional limitation, dog owners in the study group responded to a series of questions regarding weight bearing of the affected limbs; changes in posture (antalgic postures); characteristics of lameness; reluctance to move, play, or jump; and reluctance to climb stairs. The scale ranges from 0 to 23 points.

Joint mobility was measured by examining the limitations of joint movement via the range of motion of the hip joint. Maximal extension and flexion were determined using a goniometer centered on the hip center of rotation (scale 0 − 7 points). This procedure was performed on dogs from both groups by a different blinded observer (BC).

Atrophy degree was categorized as no atrophy (0 points), mild atrophy (1 point), or severe atrophy (2 points). This was performed on all dogs by the same observer. The sum of all 3 functional parameters (functional limitation, articular mobility, and muscle atrophy) categorized the arthrosis degree. A higher score indicates more severe lameness (Additional file 1).

Both VAS and Bioarth scales tests were performed at the same time as the force platform analysis test was performed.

### Statistical analysis

Parameters in this model were estimated by using the linear and nonlinear mixed effects (nlme) models package in R statistical software [34]. Data were analyzed by a different, blinded researcher who did not perform acquisition of data (AS).

A linear mixed effects model for a blocked design with repeated measures was considered. The experimental factor (time) and the status (lame–sound) of the dog were considered as fixed effects factors, while the blocking factor (dog) was a random effects factor. Because the dogs represent a random sample of the population of interest, any interaction terms modeling differences between dogs in its response when changing from different observation periods were also expressed as random effects.

Significance of the differences in PVF and VI between periods of observation were tested by means of analysis of variance of these models. Following this analysis, post-hoc comparisons between fixed effects were performed using Tukey’s procedure. For assessing the validity of the model, the Shapiro-Wilk test was applied for testing normality of the residuals. Significance level was set at *P* ≤ 0.05 in all tests.

Concordance coefficients were calculated with the F test of intraclass correlation [35] to assess the accuracy of the observer versus PVF and VI data. The irr R package was used to compute the intraclass correlation values and its significance [36]. A concordance of 1 was considered a perfect agreement, whereas a concordance of 0 referred to no agreement.

## Results

The body weight of enrolled dogs ranged from 46 to 65.2 kg (mean ± SD: 51.21 ± 5.48 kg), and ages were 4 − 9 years (mean: 5.6 ± 2.3 years). Walking velocity of both sound (control) and diseased groups of dogs was 1.6 ± 0.5 m/s. No significant difference in walking velocity was observed between dogs (*P* = 0.08).

### Force platform analysis

Mean values for PVF and VI are summarized in Table 1.

**Table 1:** Mean and standard deviation of peak vertical force (PVF) and vertical impulse (VI) in more-lame and less-lame limbs

Parameter	Day			
	0	30	90	180
PVF				
ML	39.69 ± 3.43^a^	46.73 ± 4.56^b^	41.61 ± 4.3	39.00 ± 3.82
LL	48.15 ± 6.16	49.74 ± 6.79	50.53 ± 4.7	48.13 ± 6.29
S	44.96 ± 3.67	45.91 ± 3.53	45.90 ± 3.65	45.84 ± 3.68
VI				
ML	12.16 ± 1.1^a^	14.12 ± 1.45^b^	12.58 ± 1.33	11.88 ± 1.27
LL	14.61 ± 1.9^a^	15.06 ± 2.07^b^	15.38 ± 1.55	14.66 ± 2.06
S	13.66 ± 1.28	14.04 ± 1.33	13.99 ± 1.20	14.03 ± 1.28

Results are shown in % body weight (N/N and N.s/N, respectively) for each day of observation. Different superscript letters within rows indicate significant differences (*P* < 0.05)

*ML* more-lame limbs, *LL* less-lame limbs, *S* sound limbs from control group

#### Analysis of PVF

A significant effect of time on PVF (*P* = 0.001) was detected: at day 30, more-lame limbs improved significantly (*P* = 0.0001) by 7.04 % of body weight (BW) from day 0 (Table 1). On the other hand, for days 90 and 180, there were no significant differences (*P* = 0.1573 and *P* = 0.6073, respectively) with respect to day 0.

**Fig. 2:**
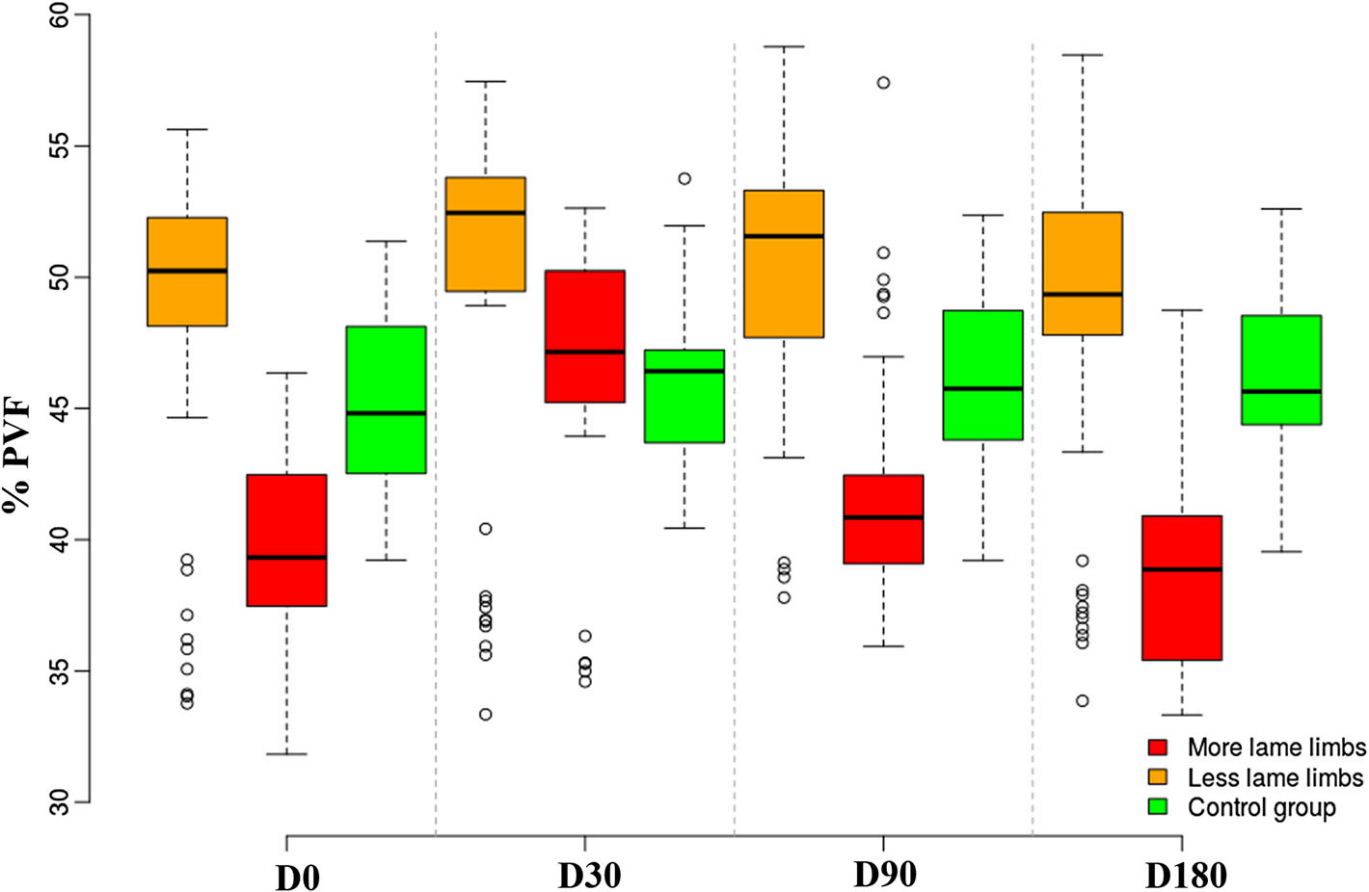
Evolution of peak vertical force (PVF) in lame-group dogs after treatment with mesenchymal stem cells at days 30, 90, and 180. The control group is also shown for reference

Compared with the control group, the day 0 lame group supported 5.28 % less BW (*P* = 0.0228). At day 30, there were no significant differences between groups (*P* = 0.6948). At day 90, the difference was still not significant (*P* = 0.0558), but at day 180, the difference (6.84 % less BW) was again significant (*P* = 0.0052) (Fig. 2).

#### Analysis of VI

**Fig. 3:**
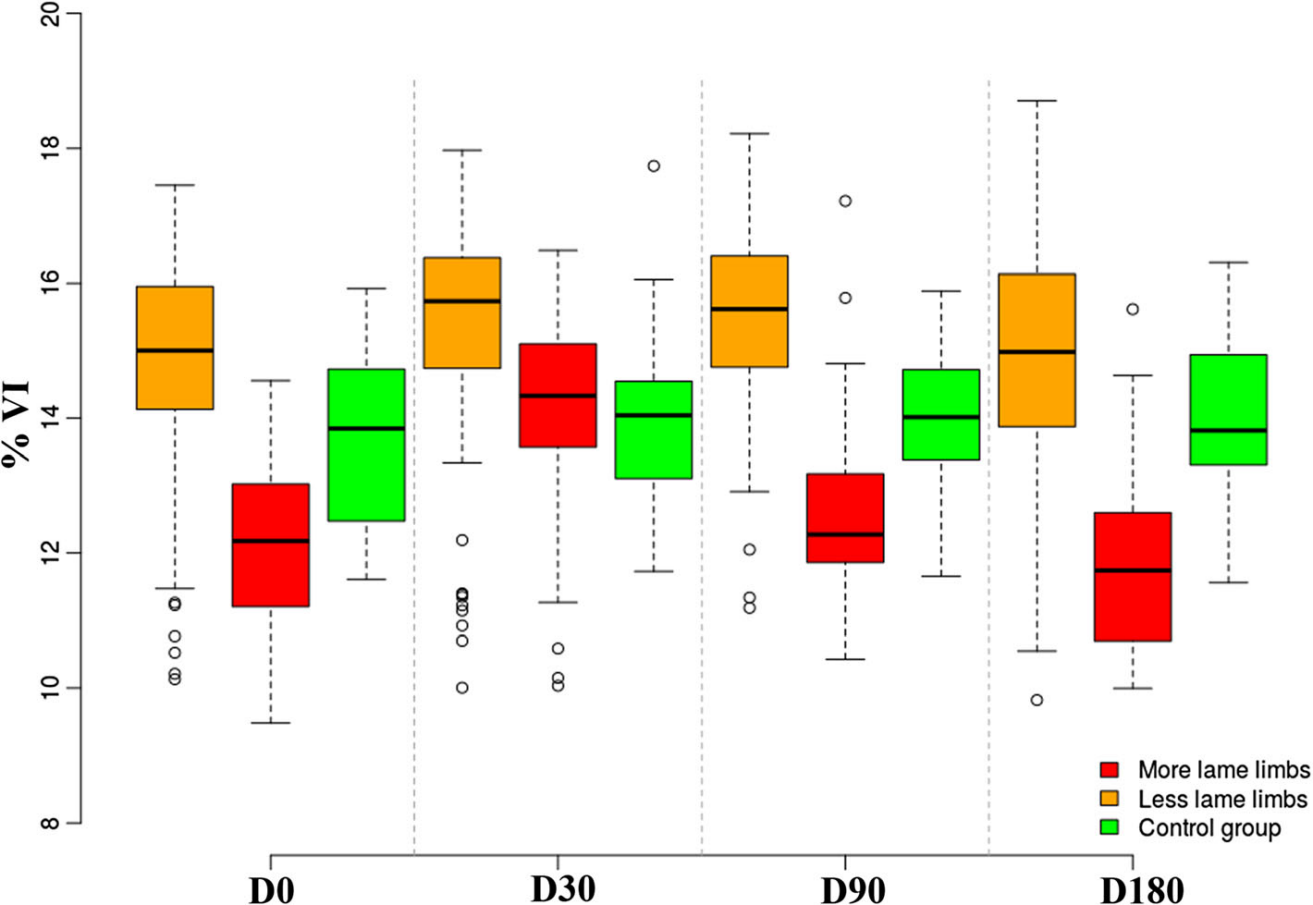
Evolution of vertical impulse (VI) in lame-group dogs after treatment with mesenchymal stem cells at days 30, 90, and 180 follow-up. The control group is also shown for reference

Compared to day 0, at day 30 there were significant differences in VI (*P* < 0.0001), but at days 90 and 180, the differences were not significant (*P* = 0.3037 and *P* = 0.4851, respectively) (Table 1). Compared with the control group, at day 0 the lame group had significant differences in VI (*P* = 0.0323). At day 30, there were no significant differences between groups (*P* = 0.9298); however, at day 90, the difference was already significant (*P* = 0.0437), and at day 180, the difference was again significant (*P* = 0.0042) (Fig. 3). The Shapiro-Wilk test showed normality of residuals (*P* = 0.6043).

#### Subjective analysis

**Fig. 4:**
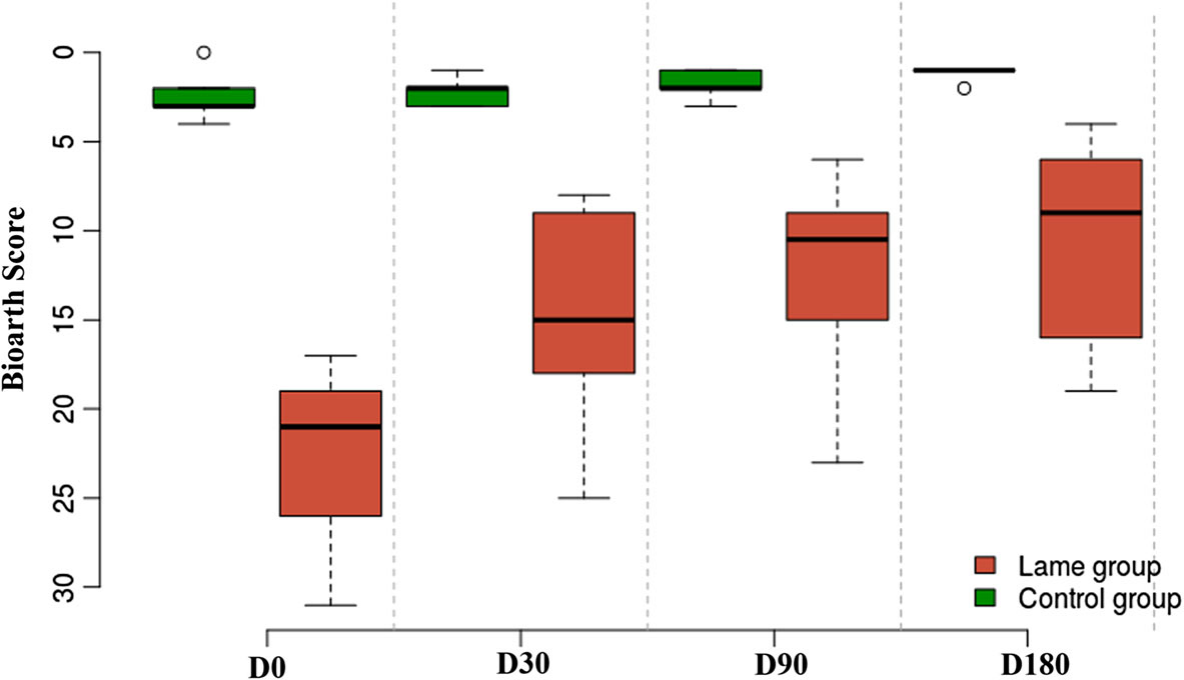
Evolution of lameness in dogs using the Bioarth scoring scale at days 0, 30, 90, and 180 post-treatment with mesenchymal stem cells. The control group is also shown for comparison

**Fig. 5:**
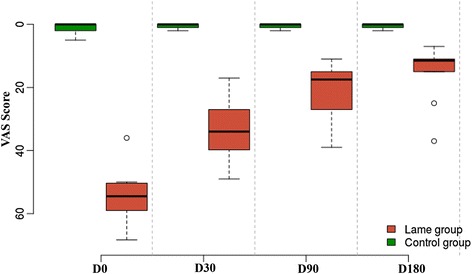
Evolution of lameness in dogs using the visual analog scoring (VAS) scale at days 0, 30, 90, and 180 post-treatment with mesenchymal stem cells. The control group is also shown for comparison

Bioarth and VAS scores are summarized in Table 2. When these results were graphically observed, scores resulting from use of both scales showed progressive improvement in lameness during the study period (Figs. 4 and 5).

**Table 2:** Mean and standard deviation of Bioarth and (VAS) scores in the treatment group

Scoring method	Day			
	0	30	90	180
Bioarth	22.3 ± 4.27	14.7 ± 5.56	11.8 ± 4.92	10.6 ± 5.27
VAS	54.77 ± 8.79	33.08 ± 9.89	20.8 ± 9.13	15.2 ± 9.03

Data are shown for each day of observation

#### Concordance analysis

Concordance values are shown in Table 3. There was a lack of concordance among objective and subjective recordings.

**Table 3:** Concordance levels among all parameters (p) used to compare subjective scale and force platform analysis of lameness scores in dogs

p1	p2	Concordance	*P* value
PVF	VI	0.996	<0.0001
PVF	BioArth	-0.504	=0.9997
PVF	VAS	-0.350	=0.9972
VI	BioArth	-0.502	=0.9641
VI	VAS	-0.354	=0.9975
BioArth	VAS	0.867	<0.0001

*PVF* peak vertical force, *VI* vertical impulse. *P* ≥ 0.05, concordance can be considered = 0. *P* < 0.05, positive concordance

## Discussion

Pain is an emotional response to a painful stimulus and is difficult to reliably determine in a nonverbal animal. Pain of the locomotor system can usually be detected by a certain disability to support weight; in other words, lameness is the expression of pain.

For clinicians, the outset of a therapeutic strategy for lameness associated with pain should be based on objective results of efficacy. This is of vital importance in order to choose the most convenient therapeutic option and to determine when a new cycle of treatment should be applied to stop a relapse [37].

In this study, the effect of MSCs on lameness in dogs affected by hip OA was investigated using subjective and objective methods. In addition, the resultant data were compared between these methods in order to determine the accuracy level of subjective methods in the evaluation of lameness in dogs.

The study was designed using a control group of sound dogs. Sound dogs were chosen as the control because, as has been previously reported, when a treated group improves its lameness, a lame, non-treated control group could worsen [38], making these animals unable to provide fixed reference data.

Patient velocity has been shown to have a significant effect on force platform values and should be limited to a narrow range when data are obtained [39]; therefore, the design for the current study included dogs of the same breed to ensure comparable conformations and a narrow range of velocities. In addition, a previous study [40] indicated that the use of multiple handlers can be an insignificant contributor of variability in a dog’s gait when narrow limits of velocity are maintained. Although the authors agree, they preferred to use the same handler for all dogs and in all testing periods in order to reach maximal uniformity.

The role of VI in measuring the evolution of lameness is controversial. While some authors suggest that recordings of improvement in VI may suffer from a delay, other authors affirm that stance time did not change or increase when limb function improved [28, 41]. In fact, the current study observed a slight difference in the evolution of both PVF and VI values; compared with sound dogs, differences in VI were already detected at day 90, while PVF did not show a return to initial state until day 180. In our opinion this happens because PVF depends only on the maximal force exerted by the limbs, while VI reflects the evolution of the force during the whole support phase. For this reason, VI could vary when one or both variables change (force and support time). Nevertheless, concordance between PVF and VI was almost 1.

Both VAS and Bioarth have been previously validated for use in assessing lameness and pain in dogs [15]. However, it has been found that these methods lack validity when performed by individuals untrained in recognizing clinical signs of pain [42]. Surprisingly, although the first part of Bioarth scoring was performed by dog owners and VAS by an experienced clinician, when these 2 scales were compared, a high concordance level was found.

When subjective and objective data were compared, concordance coefficients were calculated to assess the accuracy of the observer in the detection of variations of lameness in treated animals with respect to PVF and VI recorded from the same dogs at the same checking periods. Concordance was chosen over correlation because correlation coefficients explain how closely the variables are linearly related; however, the line they match may not have a slope of 1. Correlation disregards how close the actual data is, and instead shows how closely they fit a trend or best-fit line. Concordance coefficients explain how 2 variables are related to a line with a slope of 1. Therefore, concordance assesses how close the variables are to each other rather than the best-fit line and, in agreement with other authors, constitutes a better measure of accuracy [16, 43].

Based on the results, a great discordance was found when subjective and objective methods were compared. In fact, Bioarth and VAS scores showed good results for MSC therapy after 6 months of inoculation, in agreement with previously published data [31] (although this study had a different design and was performed with dogs of different conformations and weights). In contrast, force platform parameters demonstrated how animals had almost returned to their initial state; this discordance, in the opinion of the authors, can likely be explained by the presence of a placebo effect that affected not only the owners, but also the blind observer.

With respect to the dogs’ conformation, a molossoid breed was chosen for various reasons. Firstly, this breed shows a high incidence of hip dysplasia. Secondly, the high BW makes gait analysis easier to perform. And thirdly, this particular breed can act as an animal model potentially useful to extrapolate results to human medicine, mainly because the behavior of these animals when they are lame is similar to humans due to their heavy BW. Small and some medium dogs raise their limbs (especially pelvic limbs) to avoid pain, even when the pain is mild or moderate, making it difficult to quantify the effectiveness of a treatment. Large-breed dogs, as in humans, continue to support their lame limb/s on the ground, redistributing the weight to the contralateral limb in an effort to alleviate pain, proportionally to pain degree.

In the authors’ opinion, accurate calculation of pain (lameness) by observing certain postures, behaviors, and/or subjective appreciations is difficult, even though the observer may be an experimented clinician. In contrast, force platform analysis is more objective and quantifiable for the detection and quantification of lameness. This statement is strengthened by a previous report [1], concluding that nearly 10 times more (331 versus 38) dogs would have been needed per group to ascertain statistical significance between groups when a subjective, observed gait analysis score is used for comparison. In addition, other studies [11, 44] have previously reported poor correlation between subjective and objective methods for measuring limb function. In these studies, it was stated that a variable collected objectively and generated by the patient is superior to a variable collected subjectively and generated by an observer. For statistical interpretation, objective variables lend themselves to more powerful statistical approaches.

However, this does not necessarily mean that kinetic analysis of limb function is the only reliable indicator of the severity of pain. Objective data can also be obtained with kinematic [45] and inverse dynamics [46], avoiding the observer and inter-observer variations that are encountered with pain scoring systems.

As suggested by a recent study, the authors agree that a 6-month follow-up period can be considered a standard for testing the evolution of medical or surgical treatments [47]. In the current study, 6 months was also enough to detect great differences among different strategies to test lameness in dogs. In fact, the dogs in the current study seemed to improve during the first month after treatment, but this effect had disappeared within a period inferior to 6 months, although this change was detectable only by force platform.

In general, force platform analysis shows some limitations. First of all, much time and effort is required to obtain valid recordings. Secondly, the analysis must be performed with dogs of a relatively high body weight. Finally, these devices are relatively expensive. These considerations limit their use as ordinary diagnostic tools. On the other hand, the great shortcoming of VAS and NRS in assessing objective data from lame dogs, also supported by this study, may indicate that new subjective scoring scales should be explored if kinetic or kinematic devices for lameness detection are not available. In this line of thought, more recent studies have showed that new, validated scoring questionnaires can be reliable and give repeatable results even when used by inexperienced observers [48, 49]. Although these new tools were not used in this study, they should be explored in future studies.

## Conclusion

MSC therapy significantly improved limb function in dogs with hip OA, but the duration of the improvement was inferior to 6 months post treatment. Subjective evaluation of gait correlates poorly to objective measures of limb function. For this reason, subjective evaluation of gait should be interpreted cautiously as an outcome measure.

## Additional files

Additional file 1: List of items to score in the Bioarth scale to evaluate lameness and pain in dogs. (PDF 54 kb)
Additional file 2: Data sets. (PDF 102 kb)

## Abbreviations

BW: Body weight
i.m.: Intramuscular
i.v.: Intravenous
MSC: Mesenchymal stem cells
N: Newton
NRS: Numeric rating score
OA: Osteoarthritis
PVF: Peak vertical force
VAS: Visual analog scale
VI: Vertical impulse
