# Supplementary material for: Effect of intraarticular inoculation of mesenchymal stem cells in dogs with hip osteoarthritis by means of objective force platform gait analysis: concordance with numeric subjective scoring scales
Source: BMC Vet Res. 2016 Oct 7;12:223. doi: 10.1186/s12917-016-0852-z (PMC5055672; doi:10.1186/s12917-016-0852-z)
Supplement: Additional file 1: — List of items to score in the Bioarth scale to evaluate lameness and pain in dogs. (PDF 54 kb) [file 12917_2016_852_MOESM1_ESM.pdf]

FUNCTIONAL EVALUATION SCALE OF THE HIP

Pet Name

Owner name

A) FUNCTIONAL LIMITATION

1. CHANGES STANDING STILL: ______________________ ☐

(0) Standing normally/ (1) Leaning on one side/ (2) Resting the tip of its paw

(3) Non weight bearing on that limb

2. CHANGES STANDING UP: ______________________ ☐

(0) Standing up normally/ (1) Adopting different positions when standing up/

(2) Difficulty to rise/ (3) Does not stand up

3. LAMENESS AT THE BEGINNING OF EXERCISE: ______________ ☐

(0) No lameness present/ (1) The lameness disappears when the dog moves (up to 10 minutes)

(2) The lameness does not disappear

4. LAMENESS AFTER WARM-UP (10 MINUTES): ______________ ☐

(0) No lameness present/ (1) Mild lameness/ (2) Severe lameness

(3) Continuous non-weight-bearing lameness

5. LAMENESS DURING THE WALK: ______________________ ☐

(0) It can walk without difficulties/ (1) It often stops while going for a walk

(2) It can take just very short walks (less than 5 minutes)/

(3) It does not want to go for a walk

6. LAMENESS WHILE RUNNING AND PLAYING: ______________ ☐

(0) It can run and play without difficulties/

(1) It runs and plays with difficulties or it gets tired easily

(2) It runs with lots of difficulties under a stimulus

(3) It neither runs nor plays under any stimulus

7. GOING UP THE STEPS: ______________________ ☐

(0) It goes up without difficulties/ (1) It goes up 16 steps (a flight) with difficulty

(2) It goes up 1 or 2 steps either a kerb, with difficulty

(3) It does not go up the steps

8. SMALL JUMPS 40-50 CM: ______________________ ☐

(0) It gets on the sofa or on the car without difficulties

(1) It gets on the sofa or on the car with difficulty

(2) It neither gets on the sofa or on the car

TOTAL SCORE OF FUNCTIONAL LIMITATION ☐

(sum of scores 1-8)

FUNCTIONAL EVALUATION SCALE OF THE HIP

Pet Name

Owner name

B) RANGE OF MOVEMENT

9. MANUAL MOBILIZATION PRODUCES: ______________________________ ☐

(0) No pain and no crepitation/ (1) There is pain on the last stages

(2) There is pain and/or crepitation during the process

(3) It cannot be carried out or there is severe pain and crepitation

10. ROM IN FLEXION: ____ ☐

(0) Total flexion 50-60º/ (1) Mild limitation <80º/ (2) Severe limitation >80º

11. ROM IN EXTENSION:____ ☐

(0) Total extension 160-170º/ (1) Mild limitation>150º/ (2) Severe limitation <150º

TOTAL SCORE OF THE RANGE OF MOVEMENT ☐

(sum of scores 9-11)

C) MUSCULAR ATROPHY:____________________________________ ☐

(0) There is no muscular atrophy/ (1) Mild atrophy/ (2) Severe atrophy

TOTAL SCORE (sum of A+B+C) ☐
