# Supplementary material for: Effect of intraarticular inoculation of mesenchymal stem cells in dogs with hip osteoarthritis by means of objective force platform gait analysis: concordance with numeric subjective scoring scales
Source: BMC Vet Res. 2016 Oct 7;12:223. doi: 10.1186/s12917-016-0852-z (PMC5055672; doi:10.1186/s12917-016-0852-z)
Supplement: Additional file 2: — Data sets. (PDF 102 kb) [file 12917_2016_852_MOESM2_ESM.pdf]

dog	day	leg	diseased	weight	force	impulse
DOG-1	0	LL	TRUE	465,8	158,56	47,567
DOG-1	0	LL	TRUE	465,8	157,3	47,191
DOG-1	0	LL	TRUE	465,8	207,93	60,2984
DOG-1	0	LL	TRUE	465,8	180,96	56,0968
DOG-1	0	LL	TRUE	465,8	158,95	52,4534
DOG-1	30	LL	TRUE	465,8	176,2	52,8609
DOG-1	30	LL	TRUE	465,8	174,24	52,2723
DOG-1	30	LL	TRUE	465,8	171,85	49,8367
DOG-1	30	LL	TRUE	465,8	171,03	53,0206
DOG-1	30	LL	TRUE	465,8	188,27	62,1283
DOG-1	90	LL	TRUE	465,8	206,52	61,9559
DOG-1	90	LL	TRUE	465,8	181,07	56,1324
DOG-1	90	LL	TRUE	465,8	179,65	52,0994
DOG-1	90	LL	TRUE	465,8	182,24	60,1407
DOG-1	90	LL	TRUE	465,8	176,04	52,8106
DOG-1	180	LL	TRUE	465,8	174,43	54,0734
DOG-1	180	LL	TRUE	465,8	182,6	54,7798
DOG-1	180	LL	TRUE	465,8	176,56	58,2644
DOG-1	180	LL	TRUE	465,8	177,32	53,1945
DOG-1	180	LL	TRUE	465,8	157,75	45,7481
DOG-1	0	ML	TRUE	465,8	197,27	65,1
DOG-1	0	ML	TRUE	465,8	205,01	61,5024
DOG-1	0	ML	TRUE	465,8	179,62	53,8874
DOG-1	0	ML	TRUE	465,8	209,66	64,9951
DOG-1	0	ML	TRUE	465,8	177	51,3291
DOG-1	30	ML	TRUE	465,8	238,3	73,8731
DOG-1	30	ML	TRUE	465,8	232,37	76,6828
DOG-1	30	ML	TRUE	465,8	227,14	68,1432
DOG-1	30	ML	TRUE	465,8	245,14	73,5423
DOG-1	30	ML	TRUE	465,8	234,02	67,8661
DOG-1	90	ML	TRUE	465,8	229,94	68,9828
DOG-1	90	ML	TRUE	465,8	237,18	73,5258
DOG-1	90	ML	TRUE	465,8	267,36	80,2066
DOG-1	90	ML	TRUE	465,8	226,6	65,715
DOG-1	90	ML	TRUE	465,8	232,51	67,4279
DOG-1	180	ML	TRUE	465,8	210,23	60,9679
DOG-1	180	ML	TRUE	465,8	219,59	65,8768
DOG-1	180	ML	TRUE	465,8	227,08	68,1231
DOG-1	180	ML	TRUE	465,8	220,44	72,7445
DOG-1	180	ML	TRUE	465,8	219,84	68,1494
DOG-2	0	LL	TRUE	419,1	207,88	60,2841
DOG-2	0	LL	TRUE	419,1	210,9	65,3776
DOG-2	0	LL	TRUE	419,1	220,81	66,2418
DOG-2	0	LL	TRUE	419,1	223,57	67,0708
DOG-2	0	LL	TRUE	419,1	221,65	73,1433
DOG-2	30	LL	TRUE	419,1	215,46	66,7916
DOG-2	30	LL	TRUE	419,1	223,67	67,0997
DOG-2	30	LL	TRUE	419,1	224,72	74,1582
DOG-2	30	LL	TRUE	419,1	227,09	68,1273
DOG-2	30	LL	TRUE	419,1	222,6	64,554
DOG-2	90	LL	TRUE	419,1	215,75	66,8839
DOG-2	90	LL	TRUE	419,1	216,22	71,3535
DOG-2	90	LL	TRUE	419,1	218,5	63,3636
DOG-2	90	LL	TRUE	419,1	221,92	66,5761

DOG-2	90	LL	TRUE	419,1	227,47	68,2402
DOG-2	180	LL	TRUE	419,1	210,4	63,1191
DOG-2	180	LL	TRUE	419,1	225,11	74,2864
DOG-2	180	LL	TRUE	419,1	203,5	61,0486
DOG-2	180	LL	TRUE	419,1	220,25	63,8731
DOG-2	180	LL	TRUE	419,1	222,23	68,8908
DOG-2	0	ML	TRUE	419,1	178,67	51,8135
DOG-2	0	ML	TRUE	419,1	148,8	49,1031
DOG-2	0	ML	TRUE	419,1	155,48	46,6441
DOG-2	0	ML	TRUE	419,1	150,7	46,7161
DOG-2	0	ML	TRUE	419,1	149,49	46,3419
DOG-2	30	ML	TRUE	419,1	218,02	65,4075
DOG-2	30	ML	TRUE	419,1	194,03	60,1503
DOG-2	30	ML	TRUE	419,1	186,54	54,0967
DOG-2	30	ML	TRUE	419,1	209,34	69,0808
DOG-2	30	ML	TRUE	419,1	200,79	60,2382
DOG-2	90	ML	TRUE	419,1	156,25	46,8765
DOG-2	90	ML	TRUE	419,1	168,93	52,368
DOG-2	90	ML	TRUE	419,1	176,8	51,2732
DOG-2	90	ML	TRUE	419,1	179,15	59,1189
DOG-2	90	ML	TRUE	419,1	162,69	48,8064
DOG-2	180	ML	TRUE	419,1	148,27	44,4802
DOG-2	180	ML	TRUE	419,1	168,13	52,1201
DOG-2	180	ML	TRUE	419,1	156,17	46,8524
DOG-2	180	ML	TRUE	419,1	153,15	44,4125
DOG-2	180	ML	TRUE	419,1	159,07	52,4937
DOG-3	0	LL	TRUE	452	230,32	69,096
DOG-3	0	LL	TRUE	452	236,23	70,869
DOG-3	0	LL	TRUE	452	251,43	77,9433
DOG-3	0	LL	TRUE	452	226,68	68,004
DOG-3	0	LL	TRUE	452	241,33	74,8123
DOG-3	30	LL	TRUE	452	257,23	77,169
DOG-3	30	LL	TRUE	452	239,52	74,2512
DOG-3	30	LL	TRUE	452	245,27	73,581
DOG-3	30	LL	TRUE	452	229,76	66,6304
DOG-3	30	LL	TRUE	452	231,57	67,1553
DOG-3	90	LL	TRUE	452	238,86	71,658
DOG-3	90	LL	TRUE	452	263,23	81,6013
DOG-3	90	LL	TRUE	452	247,12	74,136
DOG-3	90	LL	TRUE	452	226,34	65,6386
DOG-3	90	LL	TRUE	452	237,35	75,952
DOG-3	180	LL	TRUE	452	219,54	65,862
DOG-3	180	LL	TRUE	452	256,21	74,3009
DOG-3	180	LL	TRUE	452	253,54	76,062
DOG-3	180	LL	TRUE	452	237,13	73,5103
DOG-3	180	LL	TRUE	452	225,75	67,725
DOG-3	0	ML	TRUE	452	166,31	49,893
DOG-3	0	ML	TRUE	452	177,82	55,1242
DOG-3	0	ML	TRUE	452	174,01	52,203
DOG-3	0	ML	TRUE	452	167,69	53,6608
DOG-3	0	ML	TRUE	452	173,05	50,1845
DOG-3	30	ML	TRUE	452	232,29	67,3641
DOG-3	30	ML	TRUE	452	223,05	66,915
DOG-3	30	ML	TRUE	452	216,19	67,0189
DOG-3	30	ML	TRUE	452	213,13	63,939

DOG-3	30	ML	TRUE	452	208,63	64,6753
DOG-3	90	ML	TRUE	452	191,48	57,444
DOG-3	90	ML	TRUE	452	179,57	55,6667
DOG-3	90	ML	TRUE	452	212,28	63,684
DOG-3	90	ML	TRUE	452	189,58	54,9782
DOG-3	90	ML	TRUE	452	193,51	59,9881
DOG-3	180	ML	TRUE	452	175,42	54,3802
DOG-3	180	ML	TRUE	452	175,9	52,77
DOG-3	180	ML	TRUE	452	184,07	55,221
DOG-3	180	ML	TRUE	452	183,88	53,3252
DOG-3	180	ML	TRUE	452	184,91	57,3221
DOG-4	0	SOUND	FALSE	475,3	193,1	57,93
DOG-4	0	SOUND	FALSE	475,3	190,52	57,156
DOG-4	0	SOUND	FALSE	475,3	195,17	60,5027
DOG-4	0	SOUND	FALSE	475,3	194,09	56,2861
DOG-4	0	SOUND	FALSE	475,3	190,22	57,066
DOG-4	30	SOUND	FALSE	475,3	196,13	60,8003
DOG-4	30	SOUND	FALSE	475,3	195,56	58,668
DOG-4	30	SOUND	FALSE	475,3	192,19	55,7351
DOG-4	30	SOUND	FALSE	475,3	193,42	58,026
DOG-4	30	SOUND	FALSE	475,3	195,19	62,4608
DOG-4	90	SOUND	FALSE	475,3	195,56	60,6236
DOG-4	90	SOUND	FALSE	475,3	191,43	57,429
DOG-4	90	SOUND	FALSE	475,3	191,03	55,3987
DOG-4	90	SOUND	FALSE	475,3	186,33	55,899
DOG-4	90	SOUND	FALSE	475,3	199,5	61,845
DOG-4	180	SOUND	FALSE	475,3	187,93	56,379
DOG-4	180	SOUND	FALSE	475,3	193,76	60,0656
DOG-4	180	SOUND	FALSE	475,3	191,23	57,369
DOG-4	180	SOUND	FALSE	475,3	189,45	54,9405
DOG-4	180	SOUND	FALSE	475,3	192,06	63,3798
DOG-5	0	LL	TRUE	509,6	184,54	57,2074
DOG-5	0	LL	TRUE	509,6	199,95	65,9835
DOG-5	0	LL	TRUE	509,6	189,19	54,8651
DOG-5	0	LL	TRUE	509,6	182,68	58,4576
DOG-5	0	LL	TRUE	509,6	178,77	53,631
DOG-5	30	LL	TRUE	509,6	183,2	56,792
DOG-5	30	LL	TRUE	509,6	191,95	55,6655
DOG-5	30	LL	TRUE	509,6	169,95	50,985
DOG-5	30	LL	TRUE	509,6	188,27	62,1291
DOG-5	30	LL	TRUE	509,6	181,54	58,0928
DOG-5	90	LL	TRUE	509,6	241,96	72,588
DOG-5	90	LL	TRUE	509,6	242,71	75,2401
DOG-5	90	LL	TRUE	509,6	243,11	75,3641
DOG-5	90	LL	TRUE	509,6	241,59	70,0611
DOG-5	90	LL	TRUE	509,6	243	75,33
DOG-5	180	LL	TRUE	509,6	189,65	56,895
DOG-5	180	LL	TRUE	509,6	183,85	56,9935
DOG-5	180	LL	TRUE	509,6	188,62	58,4722
DOG-5	180	LL	TRUE	509,6	185,34	53,7486
DOG-5	180	LL	TRUE	509,6	186,7	54,143
DOG-5	0	ML	TRUE	509,6	166,54	48,2966
DOG-5	0	ML	TRUE	509,6	179,2	53,76
DOG-5	0	ML	TRUE	509,6	162,18	53,5194
DOG-5	0	ML	TRUE	509,6	171,36	53,1216

DOG-5	0	ML	TRUE	509,6	175,46	56,1472
DOG-5	30	ML	TRUE	509,6	176,32	51,1328
DOG-5	30	ML	TRUE	509,6	180	59,4
DOG-5	30	ML	TRUE	509,6	179,83	53,949
DOG-5	30	ML	TRUE	509,6	178,37	51,7273
DOG-5	30	ML	TRUE	509,6	185,21	57,4151
DOG-5	90	ML	TRUE	509,6	213,18	61,8222
DOG-5	90	ML	TRUE	509,6	214,33	70,7289
DOG-5	90	ML	TRUE	509,6	216,39	64,917
DOG-5	90	ML	TRUE	509,6	212,6	61,654
DOG-5	90	ML	TRUE	509,6	215,65	64,695
DOG-5	180	ML	TRUE	509,6	169,83	52,6473
DOG-5	180	ML	TRUE	509,6	178,37	58,8621
DOG-5	180	ML	TRUE	509,6	175,63	50,9327
DOG-5	180	ML	TRUE	509,6	175,21	52,563
DOG-5	180	ML	TRUE	509,6	174,58	52,374
DOG-6	0	LL	TRUE	497,5	242,38	70,2894
DOG-6	0	LL	TRUE	497,5	256,23	84,5548
DOG-6	0	LL	TRUE	497,5	261,26	78,3786
DOG-6	0	LL	TRUE	497,5	256,44	76,9324
DOG-6	0	LL	TRUE	497,5	250,73	77,7274
DOG-6	30	LL	TRUE	497,5	251,15	82,8798
DOG-6	30	LL	TRUE	497,5	252,87	75,8624
DOG-6	30	LL	TRUE	497,5	260,47	80,746
DOG-6	30	LL	TRUE	497,5	263,52	76,4216
DOG-6	30	LL	TRUE	497,5	244,4	73,32
DOG-6	90	LL	TRUE	497,5	253,79	76,1381
DOG-6	90	LL	TRUE	497,5	253,13	73,4068
DOG-6	90	LL	TRUE	497,5	254,12	83,8593
DOG-6	90	LL	TRUE	497,5	252,93	73,3497
DOG-6	90	LL	TRUE	497,5	253,39	78,5509
DOG-6	180	LL	TRUE	497,5	251,28	82,9238
DOG-6	180	LL	TRUE	497,5	248,35	74,5065
DOG-6	180	LL	TRUE	497,5	245,57	73,671
DOG-6	180	LL	TRUE	497,5	231,18	67,0412
DOG-6	180	LL	TRUE	497,5	245,47	76,0971
DOG-6	0	ML	TRUE	497,5	192,1	57,6311
DOG-6	0	ML	TRUE	497,5	195,12	60,4871
DOG-6	0	ML	TRUE	497,5	192,2	55,7391
DOG-6	0	ML	TRUE	497,5	196,15	64,7308
DOG-6	0	ML	TRUE	497,5	194,33	58,3
DOG-6	30	ML	TRUE	497,5	233,32	67,6619
DOG-6	30	ML	TRUE	497,5	233,37	70,0109
DOG-6	30	ML	TRUE	497,5	231,94	71,9005
DOG-6	30	ML	TRUE	497,5	234,45	70,3347
DOG-6	30	ML	TRUE	497,5	232,33	67,3757
DOG-6	90	ML	TRUE	497,5	182,11	54,6319
DOG-6	90	ML	TRUE	497,5	179,06	51,9271
DOG-6	90	ML	TRUE	497,5	180,32	59,5047
DOG-6	90	ML	TRUE	497,5	178,87	51,8723
DOG-6	90	ML	TRUE	497,5	181,02	54,306
DOG-6	180	ML	TRUE	497,5	173,66	53,8353
DOG-6	180	ML	TRUE	497,5	175,77	50,9733
DOG-6	180	ML	TRUE	497,5	174,75	52,4263
DOG-6	180	ML	TRUE	497,5	177,39	53,2165

DOG-6	180	ML	TRUE	497,5	172,58	56,9524
DOG-7	0	LL	TRUE	529,2	257,28	74,6112
DOG-7	0	LL	TRUE	529,2	283,56	87,9036
DOG-7	0	LL	TRUE	529,2	267,55	80,265
DOG-7	0	LL	TRUE	529,2	280,87	81,4523
DOG-7	0	LL	TRUE	529,2	260,23	75,4667
DOG-7	30	LL	TRUE	529,2	258,91	75,0839
DOG-7	30	LL	TRUE	529,2	274,27	82,281
DOG-7	30	LL	TRUE	529,2	284,47	88,1857
DOG-7	30	LL	TRUE	529,2	281,96	84,588
DOG-7	30	LL	TRUE	529,2	261,78	75,9162
DOG-7	90	LL	TRUE	529,2	311,01	96,4131
DOG-7	90	LL	TRUE	529,2	275,95	88,304
DOG-7	90	LL	TRUE	529,2	250,2	75,06
DOG-7	90	LL	TRUE	529,2	228,28	73,0496
DOG-7	90	LL	TRUE	529,2	248,31	72,0099
DOG-7	180	LL	TRUE	529,2	282,58	90,4256
DOG-7	180	LL	TRUE	529,2	255,69	76,707
DOG-7	180	LL	TRUE	529,2	253,62	73,5498
DOG-7	180	LL	TRUE	529,2	229,39	73,4048
DOG-7	180	LL	TRUE	529,2	261,04	78,312
DOG-7	0	ML	TRUE	529,2	245,31	73,593
DOG-7	0	ML	TRUE	529,2	209,1	69,003
DOG-7	0	ML	TRUE	529,2	223,87	64,9223
DOG-7	0	ML	TRUE	529,2	192,6	59,706
DOG-7	0	ML	TRUE	529,2	210,94	67,5008
DOG-7	30	ML	TRUE	529,2	239,3	74,183
DOG-7	30	ML	TRUE	529,2	233,92	70,176
DOG-7	30	ML	TRUE	529,2	232,78	67,5062
DOG-7	30	ML	TRUE	529,2	235,69	70,707
DOG-7	30	ML	TRUE	529,2	232,49	72,0719
DOG-7	90	ML	TRUE	529,2	260,74	78,222
DOG-7	90	ML	TRUE	529,2	211,27	69,7191
DOG-7	90	ML	TRUE	529,2	227,47	70,5157
DOG-7	90	ML	TRUE	529,2	210,4	61,016
DOG-7	90	ML	TRUE	529,2	213,58	64,074
DOG-7	180	ML	TRUE	529,2	235,31	68,2399
DOG-7	180	ML	TRUE	529,2	211,73	69,8709
DOG-7	180	ML	TRUE	529,2	219,12	70,1184
DOG-7	180	ML	TRUE	529,2	228,82	70,9342
DOG-7	180	ML	TRUE	529,2	196,7	59,01
DOG-8	0	SOUND	FALSE	509,6	256	81,92
DOG-8	0	SOUND	FALSE	509,6	252	78,12
DOG-8	0	SOUND	FALSE	509,6	222	66,6
DOG-8	0	SOUND	FALSE	509,6	220	63,8
DOG-8	0	SOUND	FALSE	509,6	233	76,89
DOG-8	30	SOUND	FALSE	509,6	227,77	66,0533
DOG-8	30	SOUND	FALSE	509,6	240,67	79,4211
DOG-8	30	SOUND	FALSE	509,6	238,54	71,562
DOG-8	30	SOUND	FALSE	509,6	238,22	73,8482
DOG-8	30	SOUND	FALSE	509,6	236,56	70,968
DOG-8	90	SOUND	FALSE	509,6	233,13	69,939
DOG-8	90	SOUND	FALSE	509,6	230,34	71,4054
DOG-8	90	SOUND	FALSE	509,6	239,58	71,874
DOG-8	90	SOUND	FALSE	509,6	249,12	72,2448

DOG-8	90	SOUND	FALSE	509,6	242,23	79,9359
DOG-8	180	SOUND	FALSE	509,6	234,63	70,389
DOG-8	180	SOUND	FALSE	509,6	231,48	71,7588
DOG-8	180	SOUND	FALSE	509,6	243,67	73,101
DOG-8	180	SOUND	FALSE	509,6	233,87	67,8223
DOG-8	180	SOUND	FALSE	509,6	229,54	75,7482
DOG-9	0	LL	TRUE	628	337,18	101,154
DOG-9	0	LL	TRUE	628	325,43	100,8833
DOG-9	0	LL	TRUE	628	312,76	93,828
DOG-9	0	LL	TRUE	628	318,26	92,2954
DOG-9	0	LL	TRUE	628	323,91	93,9339
DOG-9	30	LL	TRUE	628	348,27	104,481
DOG-9	30	LL	TRUE	628	324,23	100,5113
DOG-9	30	LL	TRUE	628	333,67	100,101
DOG-9	30	LL	TRUE	628	338,81	105,0311
DOG-9	30	LL	TRUE	628	337,85	97,9765
DOG-9	90	LL	TRUE	628	323,28	93,7512
DOG-9	90	LL	TRUE	628	342,12	102,636
DOG-9	90	LL	TRUE	628	324,86	100,7066
DOG-9	90	LL	TRUE	628	338,21	101,463
DOG-9	90	LL	TRUE	628	332,77	96,5033
DOG-9	180	LL	TRUE	628	340,52	105,5612
DOG-9	180	LL	TRUE	628	328,88	101,9528
DOG-9	180	LL	TRUE	628	309,58	95,9698
DOG-9	180	LL	TRUE	628	320,66	96,198
DOG-9	180	LL	TRUE	628	302,62	90,786
DOG-9	0	ML	TRUE	628	241,28	72,384
DOG-9	0	ML	TRUE	628	244,37	75,7547
DOG-9	0	ML	TRUE	628	246,77	74,031
DOG-9	0	ML	TRUE	628	239,09	69,3361
DOG-9	0	ML	TRUE	628	235,25	77,6325
DOG-9	30	ML	TRUE	628	321,24	96,372
DOG-9	30	ML	TRUE	628	297,74	92,2994
DOG-9	30	ML	TRUE	628	317,08	95,124
DOG-9	30	ML	TRUE	628	329,03	95,4187
DOG-9	30	ML	TRUE	628	322,34	99,9254
DOG-9	90	ML	TRUE	628	254,32	76,296
DOG-9	90	ML	TRUE	628	274,62	79,6398
DOG-9	90	ML	TRUE	628	279,76	83,928
DOG-9	90	ML	TRUE	628	265,26	82,2306
DOG-9	90	ML	TRUE	628	256,22	81,9904
DOG-9	180	ML	TRUE	628	245,23	73,569
DOG-9	180	ML	TRUE	628	250,5	77,655
DOG-9	180	ML	TRUE	628	247,37	74,211
DOG-9	180	ML	TRUE	628	243,54	70,6266
DOG-9	180	ML	TRUE	628	246,88	76,5328
DOG-10	0	LL	TRUE	505,3	245,33	80,9594
DOG-10	0	LL	TRUE	505,3	248,12	74,4349
DOG-10	0	LL	TRUE	505,3	249,66	72,4011
DOG-10	0	LL	TRUE	505,3	235,03	72,8581
DOG-10	0	LL	TRUE	505,3	244,43	73,3286
DOG-10	30	LL	TRUE	505,3	267,31	80,1932
DOG-10	30	LL	TRUE	505,3	268,61	83,2706
DOG-10	30	LL	TRUE	505,3	259,78	77,9352
DOG-10	30	LL	TRUE	505,3	275,21	90,8182

DOG-10	30	LL	TRUE	505,3	280,25	81,2714
DOG-10	90	LL	TRUE	505,3	275,66	90,9686
DOG-10	90	LL	TRUE	505,3	254,67	78,9488
DOG-10	90	LL	TRUE	505,3	272,25	78,9538
DOG-10	90	LL	TRUE	505,3	264,42	79,3268
DOG-10	90	LL	TRUE	505,3	251,52	75,4573
DOG-10	180	LL	TRUE	505,3	244,04	80,5332
DOG-10	180	LL	TRUE	505,3	260,44	78,1333
DOG-10	180	LL	TRUE	505,3	245,75	71,2675
DOG-10	180	LL	TRUE	505,3	251,99	75,5977
DOG-10	180	LL	TRUE	505,3	252,17	78,1713
DOG-10	0	ML	TRUE	505,3	222,87	73,5486
DOG-10	0	ML	TRUE	505,3	222,78	66,8355
DOG-10	0	ML	TRUE	505,3	222,54	66,7611
DOG-10	0	ML	TRUE	505,3	222,62	69,0131
DOG-10	0	ML	TRUE	505,3	222,77	64,6038
DOG-10	30	ML	TRUE	505,3	228,29	70,7692
DOG-10	30	ML	TRUE	505,3	228,5	66,2643
DOG-10	30	ML	TRUE	505,3	228,74	68,6211
DOG-10	30	ML	TRUE	505,3	228,52	68,556
DOG-10	30	ML	TRUE	505,3	228,23	66,1867
DOG-10	90	ML	TRUE	505,3	189,76	56,9279
DOG-10	90	ML	TRUE	505,3	189,81	62,6377
DOG-10	90	ML	TRUE	505,3	190,3	55,1861
DOG-10	90	ML	TRUE	505,3	189,44	56,8313
DOG-10	90	ML	TRUE	505,3	189,69	58,805
DOG-10	180	ML	TRUE	505,3	178,99	51,9074
DOG-10	180	ML	TRUE	505,3	178,64	53,5918
DOG-10	180	ML	TRUE	505,3	179,13	53,7376
DOG-10	180	ML	TRUE	505,3	178,97	59,0595
DOG-10	180	ML	TRUE	505,3	178,74	51,8346
DOG-11	0	SOUND	FALSE	470,4	224,49	67,347
DOG-11	0	SOUND	FALSE	470,4	235,61	70,683
DOG-11	0	SOUND	FALSE	470,4	242,78	72,834
DOG-11	0	SOUND	FALSE	470,4	230,99	71,6069
DOG-11	0	SOUND	FALSE	470,4	211,91	67,8112
DOG-11	30	SOUND	FALSE	470,4	220,56	66,168
DOG-11	30	SOUND	FALSE	470,4	217,76	67,5056
DOG-11	30	SOUND	FALSE	470,4	252,87	83,4471
DOG-11	30	SOUND	FALSE	470,4	238,56	69,1824
DOG-11	30	SOUND	FALSE	470,4	224,34	67,302
DOG-11	90	SOUND	FALSE	470,4	246,26	73,878
DOG-11	90	SOUND	FALSE	470,4	243,12	72,936
DOG-11	90	SOUND	FALSE	470,4	218,22	63,2838
DOG-11	90	SOUND	FALSE	470,4	229,24	71,0644
DOG-11	90	SOUND	FALSE	470,4	226,46	74,7318
DOG-11	180	SOUND	FALSE	470,4	247,43	76,7033
DOG-11	180	SOUND	FALSE	470,4	234,25	70,275
DOG-11	180	SOUND	FALSE	470,4	213,34	64,002
DOG-11	180	SOUND	FALSE	470,4	228,34	66,2186
DOG-11	180	SOUND	FALSE	470,4	230,56	76,0848
DOG-12	0	SOUND	FALSE	490	223,54	67,062
DOG-12	0	SOUND	FALSE	490	225,55	69,9205
DOG-12	0	SOUND	FALSE	490	216,49	64,947
DOG-12	0	SOUND	FALSE	490	214,98	62,3442

DOG-12	0	SOUND	FALSE	490	212,64	70,1712
DOG-12	30	SOUND	FALSE	490	213,8	64,14
DOG-12	30	SOUND	FALSE	490	218,28	67,6668
DOG-12	30	SOUND	FALSE	490	228,2	68,46
DOG-12	30	SOUND	FALSE	490	222,73	71,2736
DOG-12	30	SOUND	FALSE	490	229,28	68,784
DOG-12	90	SOUND	FALSE	490	218,58	65,574
DOG-12	90	SOUND	FALSE	490	224,3	67,29
DOG-12	90	SOUND	FALSE	490	216,22	67,0282
DOG-12	90	SOUND	FALSE	490	223,4	64,786
DOG-12	90	SOUND	FALSE	490	222,64	71,2448
DOG-12	180	SOUND	FALSE	490	217,46	67,4126
DOG-12	180	SOUND	FALSE	490	223,65	67,095
DOG-12	180	SOUND	FALSE	490	223,87	64,9223
DOG-12	180	SOUND	FALSE	490	221,98	73,2534
DOG-12	180	SOUND	FALSE	490	221,19	66,357
DOG-13	0	LL	TRUE	480	261,25	78,375
DOG-13	0	LL	TRUE	480	247,23	76,6413
DOG-13	0	LL	TRUE	480	247,22	81,5826
DOG-13	0	LL	TRUE	480	238,16	71,448
DOG-13	0	LL	TRUE	480	236,75	68,6575
DOG-13	30	LL	TRUE	480	274,51	85,0981
DOG-13	30	LL	TRUE	480	245,17	73,551
DOG-13	30	LL	TRUE	480	247,27	71,7083
DOG-13	30	LL	TRUE	480	258,67	77,601
DOG-13	30	LL	TRUE	480	262	78,6
DOG-13	90	LL	TRUE	480	266,45	79,935
DOG-13	90	LL	TRUE	480	236,23	73,2313
DOG-13	90	LL	TRUE	480	255,87	76,761
DOG-13	90	LL	TRUE	480	250,74	72,7146
DOG-13	90	LL	TRUE	480	260,37	83,3184
DOG-13	180	LL	TRUE	480	235,34	70,602
DOG-13	180	LL	TRUE	480	247,21	76,6351
DOG-13	180	LL	TRUE	480	229,43	66,5347
DOG-13	180	LL	TRUE	480	256,87	77,061
DOG-13	180	LL	TRUE	480	266,55	85,296
DOG-13	0	ML	TRUE	480	199,84	59,952
DOG-13	0	ML	TRUE	480	190,88	59,1728
DOG-13	0	ML	TRUE	480	203,29	60,987
DOG-13	0	ML	TRUE	480	206	59,74
DOG-13	0	ML	TRUE	480	198,56	65,5248
DOG-13	30	ML	TRUE	480	244,26	75,7206
DOG-13	30	ML	TRUE	480	231,68	67,1872
DOG-13	30	ML	TRUE	480	232,74	69,822
DOG-13	30	ML	TRUE	480	241,62	72,486
DOG-13	30	ML	TRUE	480	244,47	73,341
DOG-13	90	ML	TRUE	480	198,07	59,421
DOG-13	90	ML	TRUE	480	187,59	58,1529
DOG-13	90	ML	TRUE	480	193,71	58,113
DOG-13	90	ML	TRUE	480	193,61	56,1469
DOG-13	90	ML	TRUE	480	197,67	57,3243
DOG-13	180	ML	TRUE	480	187,32	56,196
DOG-13	180	ML	TRUE	480	182,15	56,4665
DOG-13	180	ML	TRUE	480	184,43	55,329
DOG-13	180	ML	TRUE	480	195,27	56,6283

DOG-13	180	ML	TRUE	480	180,05	57,616
DOG-14	0	LL	TRUE	464	246,75	74,025
DOG-14	0	LL	TRUE	464	237,96	71,388
DOG-14	0	LL	TRUE	464	245,21	71,1109
DOG-14	0	LL	TRUE	464	223,36	69,2416
DOG-14	0	LL	TRUE	464	215,31	62,4399
DOG-14	30	LL	TRUE	464	266,54	79,962
DOG-14	30	LL	TRUE	464	243,74	75,5594
DOG-14	30	LL	TRUE	464	257,23	77,169
DOG-14	30	LL	TRUE	464	238,56	69,1824
DOG-14	30	LL	TRUE	464	246,1	71,369
DOG-14	90	LL	TRUE	464	252,86	78,3866
DOG-14	90	LL	TRUE	464	261,23	78,369
DOG-14	90	LL	TRUE	464	241,44	72,432
DOG-14	90	LL	TRUE	464	239,07	69,3303
DOG-14	90	LL	TRUE	464	240,6	76,992
DOG-14	180	LL	TRUE	464	256,23	76,869
DOG-14	180	LL	TRUE	464	224,76	69,6756
DOG-14	180	LL	TRUE	464	249,54	74,862
DOG-14	180	LL	TRUE	464	233,32	67,6628
DOG-14	180	LL	TRUE	464	271,25	86,8
DOG-14	0	ML	TRUE	464	197,1	59,13
DOG-14	0	ML	TRUE	464	206,03	61,809
DOG-14	0	ML	TRUE	464	187,46	54,3634
DOG-14	0	ML	TRUE	464	194,95	60,4345
DOG-14	0	ML	TRUE	464	197,37	63,1584
DOG-14	30	ML	TRUE	464	218,76	65,628
DOG-14	30	ML	TRUE	464	212,27	65,8037
DOG-14	30	ML	TRUE	464	224,02	67,206
DOG-14	30	ML	TRUE	464	237,43	68,8547
DOG-14	30	ML	TRUE	464	224,31	71,7792
DOG-14	90	ML	TRUE	464	193,02	57,906
DOG-14	90	ML	TRUE	464	188,97	58,5807
DOG-14	90	ML	TRUE	464	183,65	55,095
DOG-14	90	ML	TRUE	464	189,76	55,0304
DOG-14	90	ML	TRUE	464	188,11	56,433
DOG-14	180	ML	TRUE	464	194,87	58,461
DOG-14	180	ML	TRUE	464	189,96	56,988
DOG-14	180	ML	TRUE	464	191,34	59,3154
DOG-14	180	ML	TRUE	464	187,36	54,3344
DOG-14	180	ML	TRUE	464	195,43	60,5833
DOG-15	0	SOUND	FALSE	450,8	224,71	67,413
DOG-15	0	SOUND	FALSE	450,8	219,57	63,6753
DOG-15	0	SOUND	FALSE	450,8	214,3	62,147
DOG-15	0	SOUND	FALSE	450,8	212,36	61,5844
DOG-15	0	SOUND	FALSE	450,8	236,24	73,2344
DOG-15	30	SOUND	FALSE	450,8	198,95	59,685
DOG-15	30	SOUND	FALSE	450,8	227,21	70,4351
DOG-15	30	SOUND	FALSE	450,8	196,93	59,079
DOG-15	30	SOUND	FALSE	450,8	219,36	72,3888
DOG-15	30	SOUND	FALSE	450,8	234,22	67,9238
DOG-15	90	SOUND	FALSE	450,8	232,56	69,768
DOG-15	90	SOUND	FALSE	450,8	196,33	60,8623
DOG-15	90	SOUND	FALSE	450,8	221,16	66,348
DOG-15	90	SOUND	FALSE	450,8	223,34	64,7686

DOG-15	90	SOUND	FALSE	450,8	197,42	65,1486
DOG-15	180	SOUND	FALSE	450,8	232,24	69,672
DOG-15	180	SOUND	FALSE	450,8	219,46	68,0326
DOG-15	180	SOUND	FALSE	450,8	198,25	59,475
DOG-15	180	SOUND	FALSE	450,8	229,87	66,6623
DOG-15	180	SOUND	FALSE	450,8	218,23	72,0159

dog	diseased	day	LIMITAC.FUN	MOV.ARTIC	ATROFIA	BIOARTH.SC	VAS.SCORE
DOG-1	TRUE	0	10	6	1	17	50
DOG-1	TRUE	30	3	3	2	8	35
DOG-1	TRUE	90	3	2	1	6	18
DOG-1	TRUE	180	1	1	2	4	10
DOG-2	TRUE	0	16	7	3	26	53
DOG-2	TRUE	30	10	5	3	18	27
DOG-2	TRUE	90	7	6	2	15	15
DOG-2	TRUE	180	11	3	2	16	12
DOG-3	TRUE	0	13	4	2	19	58
DOG-3	TRUE	30	7	1	1	9	20
DOG-3	TRUE	90	6	2	2	10	17
DOG-3	TRUE	180	4	1	1	6	11
DOG-4	FALSE	0	0	1	2	3	5
DOG-4	FALSE	30	0	2	1	3	2
DOG-4	FALSE	90	0	1	1	2	2
DOG-4	FALSE	180	0	0	2	2	2
DOG-5	TRUE	0	17	7	2	26	68,33
DOG-5	TRUE	30	17	5	3	25	39,78
DOG-5	TRUE	90	13	7	3	23	27
DOG-5	TRUE	180	8	6	2	16	15
DOG-6	TRUE	0	16	4	2	22	64
DOG-6	TRUE	30	9	6	2	17	49
DOG-6	TRUE	90	6	5	1	12	39
DOG-6	TRUE	180	13	4	2	19	37
DOG-7	TRUE	0	23	6	2	31	59
DOG-7	TRUE	30	12	4	2	18	17
DOG-7	TRUE	90	5	5	1	11	11
DOG-7	TRUE	180	4	5	1	10	7
DOG-8	FALSE	0	1	2	1	4	2
DOG-8	FALSE	30	1	1	0	2	1
DOG-8	FALSE	90	1	2	0	3	1
DOG-8	FALSE	180	1	0	0	1	1
DOG-9	TRUE	0	14	5	1	20	50,33
DOG-9	TRUE	30	6	1	2	9	31
DOG-9	TRUE	90	6	2	1	9	11
DOG-9	TRUE	180	6	2	0	8	11
DOG-10	TRUE	0	16	6	1	23	54
DOG-10	TRUE	30	14	1	4	19	36
DOG-10	TRUE	90	10	3	2	15	23
DOG-10	TRUE	180	10	3	1	14	13
DOG-11	FALSE	0	0	2	0	2	0
DOG-11	FALSE	30	0	2	0	2	0
DOG-11	FALSE	90	0	1	0	1	0
DOG-11	FALSE	180	0	1	0	1	0
DOG-12	FALSE	0	0	0	0	0	0
DOG-12	FALSE	30	1	0	0	1	0
DOG-12	FALSE	90	0	1	0	1	0
DOG-12	FALSE	180	0	1	0	1	0
DOG-13	TRUE	0	15	4	1	20	36
DOG-13	TRUE	30	9	3	1	13	33
DOG-13	TRUE	90	5	1	1	7	16
DOG-13	TRUE	180	4	1	0	5	11
DOG-14	TRUE	0	12	6	1	19	55
DOG-14	TRUE	30	6	3	2	11	43
DOG-14	TRUE	90	4	5	1	10	31
DOG-14	TRUE	180	4	3	1	8	25
DOG-15	FALSE	0	0	2	1	3	0
DOG-15	FALSE	30	1	2	0	3	0
DOG-15	FALSE	90	0	1	1	2	0
DOG-15	FALSE	180	0	0	1	1	0
